# Stearoyl-Chitosan Coated Nanoparticles Obtained by Microemulsion Cold Dilution Technique

**DOI:** 10.3390/ijms19123833

**Published:** 2018-11-30

**Authors:** Daniela Chirio, Elena Peira, Simona Sapino, Chiara Dianzani, Alessandro Barge, Elisabetta Muntoni, Silvia Morel, Marina Gallarate

**Affiliations:** 1 University of Turin, Dipartimento di Scienza e Tecnologia del Farmaco, via Giuria 9, Torino 10125, Italy; daniela.chirio@unito.it (D.C.); chiara.dianzani@unito.it (C.D.); alessandro.barge@unito.it (A.B.); elisabetta.muntoni@unito.it (E.M.); marina.gallarate@unito.it (M.G.); 2 Amedeo Avogadro University of Eastern Piedmont, Dipartimento di Scienze del Farmaco, Largo Donegani 2/3, Novara 28100, Italy; silvia.morel@uniupo.it

**Keywords:** nanoparticle, chitosan, microemulsion, curcumin, biodistribution, cancer

## Abstract

Chitosan is an excipient which has been studied thoroughly in research works thanks to its positive characteristics such as muco-adhesiveness and ability to open epithelial-tight-junctions. In this article, lipophilic stearoyl chitosan (ST-CS) was synthetized in order to anchor this polymer to lipid nanoparticles and prepare ST-CS-coated nanoparticles (ST-CS-NP) using the microemulsion cold dilution technique. Curcumin (CURC) was used as model drug. CURC-ST-CS-NP were characterized by dimensional analysis, zeta potential, drug entrapment, drug release; tested in vitro on Human Umbilical Vein Endothelial Cell (HUVEC) cells to study its cytotoxicity and on human pancreatic cancer cells (PANC-1) to determine inhibition ability; tested in rats to determine CURC blood profiles and biodistribution. CURC-ST-CS-NP had mean diameters in the range 200–400 nm and CURC entrapment up to 73%. These systems did not show cytotoxicity on HUVEC cells at all tested dilutions and revealed to be more effective than free CURC solution on PANC-1 cells at 5 and 10 µM CURC. Blood profile studies evidenced as CURC entrapment in NP prolonged the permanence of drug in the systemic circulation compared to CURC solution due to a certain stealth property of NP, probably attributable to hydrophilic chitosan coating. Biodistribution studies showed a smaller CURC concentration in RES organs when CURC-ST-CS-NP were administered.

## 1. Introduction

Chitosan (CS) is a linear copolymer of β-(1–4)-linked d-glucosamine and N-acetyl-d-glucosamine with hydrophilic characteristics and cationic character due to its primary amino groups. The presence of amino groups, protonated in physiological fluids, imparts interesting biopharmaceutical properties to CS, such as muco-adhesiveness and ability to open epithelial tight junctions: in fact, it seems that the polymer positive charges interact with the cell membranes resulting in a structural reorganization of tight junction-associated proteins [1].

These characteristics, together with its well-known low toxicity, excellent biocompatibility and biodegradability, have attracted the attention of many researchers in biomedical and in drug delivery fields [2].

Many CS-based nanocarriers have been studied for a variety of applications such as topical ocular and dermal drug delivery, transmucosal, oral/nasal peptide absorption, anti-cancer drug delivery, brain delivery and gene delivery [3]. 

Owing to its hydrophilic character, in drug delivery it is often necessary to use crosslinking agents such as tripolyphosphate and glutaraldehyde or to synthesize CS amphiphilic derivatives exploiting the chemically reactive amino and hydroxyl groups [4,5]. 

Fan et al. [6] used tripolyphosphate as crosslinking agent to form CS nanoparticles. In their work they established that the concentration of acetic acid used to dissolve CS and the temperature at which the cross-linking process occurs strongly affect the polydispersion of the obtained nanoparticles. They demonstrated that by performing the reaction below 4 °C, it was possible to obtain a polydispersity index below 0.05, probably as the reduced temperature increases the number of hydrogen bonds between the polar groups of CS and the surrounding water, leading to the formation of a hydration layer around the nanoparticles, which decreased the probability of nanoparticle collision.

Qu et al. [7] prepared Fe_3_O_4_–CS nanoparticles using glutaraldehyde as crosslinking agent; these nanoparticles showed a highly saturated magnetization effect, superparamagnetic properties and a sufficiently high temperature to induce hyperthermia.

Anyway, cross-linkers such as glutaraldehyde were found to cause overt toxicity and to compromise drug integrity [8]. For these reasons, the focus of the researchers on the use of CS nanoparticles was progressively shifted toward less aggressive procedures such as the synthesis of CS amphiphilic derivatives. 

Peng et al. [9] synthesized a novel amphiphilic CS derivative (N,N-dimethylhexadecyl carboxymethyl CS) to prepare salidroside-loaded liposomes. These carriers, compared with traditional liposomes (phosphatidylcholine/cholesterol liposomes), showed higher encapsulation efficiency and slower sustained release rate, resulting suitable for the sustained release of salidroside.

Amphiphilic N-Succinyl-N′-octyl CS was synthesized by Xiangyang et al. [10]. This polymer forms micelles in water, able to load doxorubicin. They found that the loading amount of doxorubicin increased with increasing drug-to-carrier ratio, and when larger amount of the octyl chain was used, higher drug loading was obtained. Doxorubicin-loaded polymeric micelles, containing the drug in the core region) exhibited sustained release and more cytotoxic activity against HepG2, A549, BGC and K562 than doxorubicin alone.

In the present work, to avoid the toxicity of most crosslinker agents and to anchor CS more firmly to lipid nanoparticles (NP), amphiphilic stearoyl-CS (ST-CS) was synthesized. The aim was to obtain a CS derivative able to be used with lecithin and cholesterol as lipid matrix in NP formulation. It is assumed that, thanks to its amphiphilic nature, ST-CS can locate inside NP with its lipophilic chains and put the hydrophilic hydroxy groups outward. In this way, ST-CS will confer hydrophilic character to the NP, useful requirement to prolong plasma half-life after in vivo administration. The lipophilic stearoyl chains, embedded in the lipid matrix of NP, will ensure a stronger and longer interaction of the CS moiety with the lipid matrix, other than a simple surface adsorption. 

Curcumin (CURC)-loaded ST-CS NP (CURC-ST-CS-NP) were prepared with the innovative technique called “cold dilution of microemulsion”. This technique, unlike most methods described in the literature, does not require high temperatures, sonication, pH variations that could negatively influence drug stability or entrapment. It combines the advantages of an emulsion solvent diffusion technique with the high stability and the super solvent properties of microemulsive systems. This method does not require low or high temperatures, other than the patented method of Gasco [11] which employed a molten lipid substance added to a hot aqueous surfactant–cosurfactant dispersion. 

CURC, a natural polyphenol, was chosen as for its multiple actions such as antioxidant, anti-inflammatory properties, it plays a significant beneficial and pleiotropic regulatory role in various pathological conditions including cancer, cardiovascular disease, Alzheimer’s disease, inflammatory disorders, neurological disorders [12]. When used in combination, CURC has also been shown to potentiate the effects of other cytotoxic agents, including gemcitabine, cisplatin, oxaliplatin, and 5-fluorouracil, in preclinical models of a variety of cancers. A recent study demonstrated CURC restores sensitivity in gemcitabine-resistant cancer cells and confirmed this finding in a xenograft mouse model. (Stephen Bigelsen, “Evidence-based complementary treatment of pancreatic cancer: a review of adjunct therapies including paricalcitol, hydroxychloroquine, intravenous vitamin C, statins, metformin, curcumin, and aspirin Cancer Manag Res. 2018; 10: 2003–2018.”) Disappointingly, its low bioavailability limits its effectiveness. To improve the bioavailability of CURC, numerous approaches have been undertaken.

In a previous work [13] we noted that CURC photostability and hydrolytic stability were significantly enhanced when it was entrapped in triglicerides lipid nanoparticles. Later, CURC-loaded solid lipid nanoparticles of several fatty acids were prepared with the coacervation technique and CS hydrochloride was added to produce bioadhesive, positively-charged nanoparticles [14]. Generally, spherical shaped nanoparticles with mean diameters below 500 nm were obtained, in which CURC entrapment efficiency was in the 28–81% range and was highly influenced by both FA and polymer type.

By combining the cold dilution of microemulsions technique with the use of stearoyl CS, we hope to obtain biocompatible CURC-loaded NP with high entrapment efficiency to be tested in vitro on PANC-1 cells for cytotoxicity and in vivo in biodistribution studies.

## 2. Results and Discussion

CS, a polysaccharide with cationic character due to its primary amino groups, is an excipient widely used in drug delivery for its positive properties such as the ability to provide controlled drug release, mucoadhesivity, in situ gelation, transfection, permeation enhancement and for its efflux pump inhibitory properties [1].

To take advantage from these properties, in this experimental work lipophilic NP prepared with biocompatible ingredients were coated with CS. 

Preliminary studies (data not shown) were carried out using commercial CS crosslinked with glutaraldehyde or sodium tripolyphosphate. Such coating proved to be unsuitable for these systems, as a significant loss of adhesion to NP was noted as a consequence of nanoparticle suspension dilution.

In this paper, an experimental work is described in which a lipophilic CS derivative was synthesized using stearoyl chloride as esterifying agent; the lipophilic C18 chains, linked to CS amine groups through amidic bonds, might anchor this coating to the lipid NP matrix.

**Figure 1:**
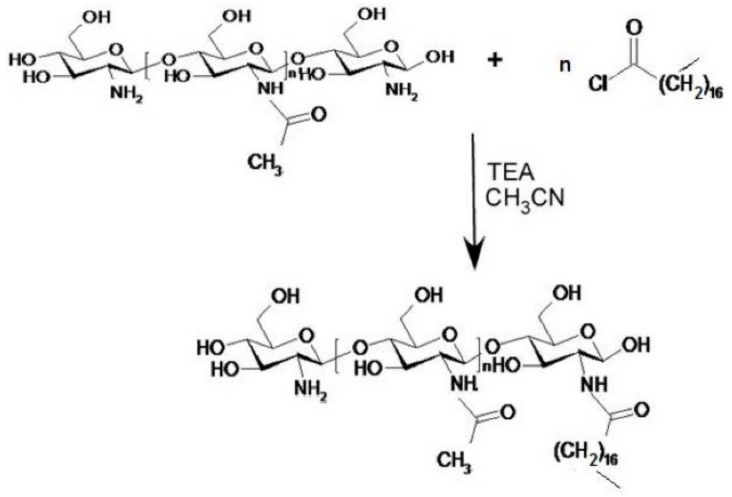
Stearoyl chitosan (ST-CS) synthesis scheme. Chitosan (CS) and different amounts of stearoyl chloride (corresponding to 30% and 50% of the number of amino groups present on native CS) were added to CH_3_CN and triethylamine and kept in an ultrasound bath at 25 °C for 4 h.

Some ST-CS samples with different degree of substitution were prepared using an ultrawave technique according to the reaction scheme reported in Figure 1.

**Figure 2:**
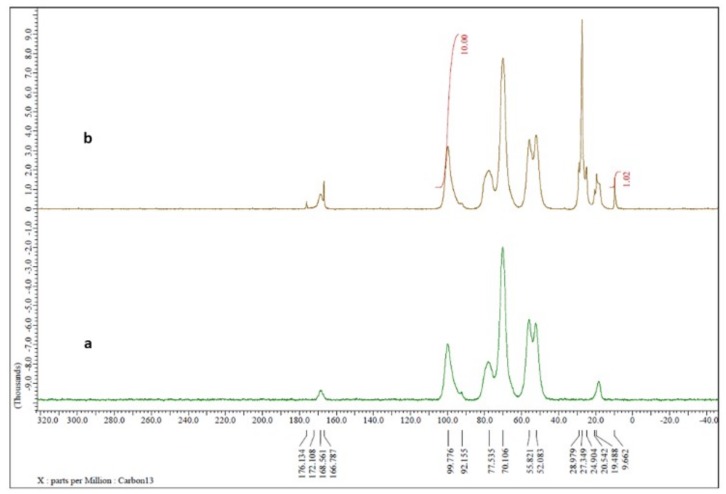
CS (**a**) and ST-CS (**b**) nuclear magnetic resonance (NMR) spectra performed using Jeol ECZ-R 600 MHz spectrometer equipped with a solid probe operating at 14 T at room temperature and spectral width of 10 KHz.

Reaction yields were about 90% for all samples that were then characterized by nuclear magnetic resonance (NMR) analysis. Due to the poor solubility of ST-CS in solvents normally used in NMR analysis, spectra were recorded directly on the freeze-dried powder. Comparing the commercial CS NMR spectrum (Figure 2a) with the spectrum of ST-CS obtained with lower amount of stearoyl chloride (Figure 2b), a series of peaks in the 10–30 ppm range was present in the latter spectrum, signals attributable to alkyl chain carbons. 

By integration of CS anomeric carbon peak at 100 ppm and of that of methyl carbon alkyl chain at 10 ppm, it seems that CS with 10% and 20% of substituted amine groups instead of theoretical 30% and 50% was obtained, respectively. 

NP were prepared using a new technique called “cold dilution of microemulsion”, an innovative method based on the use of a partially water-soluble organic solvent as µE disperse phase. Owing to µE water-dilution at room temperature, the organic solvent of the internal phase diffuses into the added water determining the precipitation of lipids as NP. This technique presents many advantages: it does not require high operating temperatures; because of its relative simplicity no complex equipment is required; the high contents of surfactants/co-surfactants in the microemulsion (µE) allow to entrap significant amounts of drug, due to their solubilizing capacity. As a consequence, also µE-derived NP will be able to exert a fair drug loading.

Following a preliminary formulation study to screen several excipients and organic solvents suitable to form a µE, lecithin and cholesterol (CHOL) were chosen as biocompatible lipid NP matrices and water-saturated butyl lactate (s-BL) as organic internal phase. Some µE were formulated with different surfactant/co-surfactant (Table 1) chosen among those accepted for parenteral administration. 

**Figure 3:**
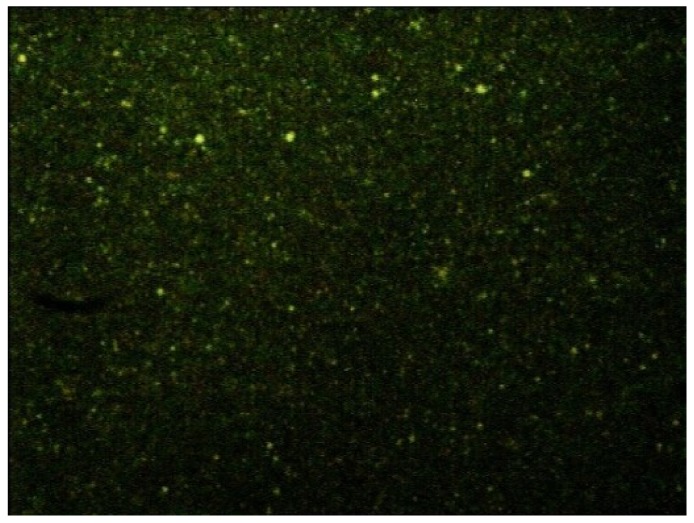
Micrograph of fluorescein isothiocyanate (FITC)-ST-CS NP observed by optical microscope equipped with fluorescent lamp at 787× magnification: fluorescence is due to ST-CS-FITC.

Preliminary NP formulation studies with both 10% and 20% substituted CS were performed; because particles with larger sizes and less stability have been obtained with the less lipophilic derivative (data not shown), 20% substituted CS (ST-CS, from here onwards) was chosen to continue the study. To verify the localization of the CS coating, ST-CS was made fluorescent by reaction with fluorescein isothiocyanate (FITC); a µE containing ST-CS-FITC was prepared, diluted, purified and the resulting nanoparticles observed by optical microscope equipped with fluorescent lamp. In Figure 3, a micrograph of such NP is reported. 

As the fluorescence is due only to ST-CS-FITC and as this remained also after nanoparticle purification by gel chromatography, ST-CS was probably located around NP and anchored to their matrix: the small size of nanoparticles allows only optical microscope images appearing like fluorescent points to be obtained. The presence of a fluorescent CS allowed us both to identify its location on NP surface and to evidence NP size, which, being in the 200–400 nm range, lies around the optical microscopy detection limit. To obtain a more accurate NP size evaluation, mean diameters of all prepared nanoparticles were determined by dynamic light scattering (DLS)

A series of drug-free µE (µE 1–5) was prepared to set up the new preparation technique and to test the influence of different surfactant mixtures on the resulting NP. Looking at mean diameters reported in Table 2, it seems that Cremophor^®^RH60/TC sodium salt was the surfactant mixture that allowed to obtain the smallest NP (NP-4). On the other hand, the presence of PEG 400 determined a certain NP size increase depending on its concentration. 

The same µE were also prepared in the presence of CURC, a molecule widely studied for its promising biological and pharmacological activities [15] but with great bioavailability problems [16].

CURC-ST-CS-NP, obtained by water dilution, have mean diameters similar to CURC-free ones: similarly, the use of Cremophor^®^RH60/TC sodium salt mixture allowed us to obtain the smallest NP, while the presence of PEG 400 determined an increase in size. Also, CURC EE% was influenced by the surfactant/cosurfactant mixture used: the best result obtained was 73.4% in CURC-NP-9 prepared starting from µE-9 containing Cremophor^®^RH60/TC sodium salt mixture. Zeta potential determination showed a slight positive value for all formulations, probably due to CS amino groups that are partially protonated in the phosphate-buffered saline (PBS) used in NP purification. The presence of CURC seems to have no influence. NP-4 and CURC-NP-9 showed the lowest mean diameters and, therefore, they were chosen for further studies on cell lines and for in vivo administration in rats.

**Figure 4:**
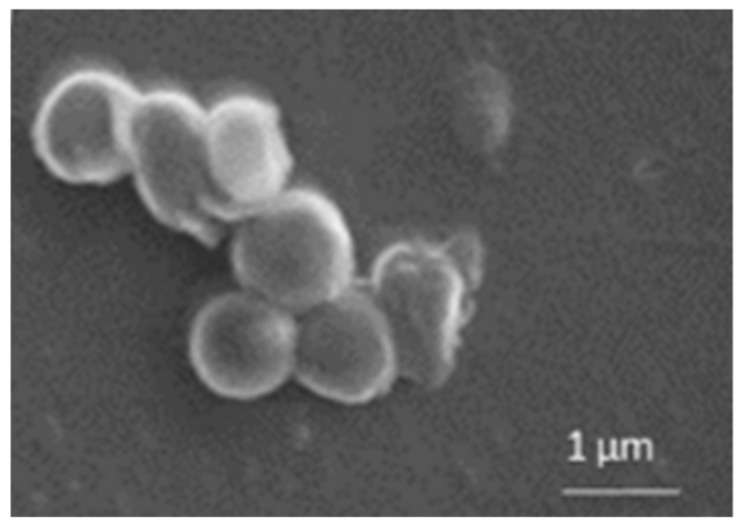
Scanning electron microscope (SEM) micrograph of NP-4 observed at 20,000 X magnification.

To confirm the size of nanoparticles determined by DLS measurement, and to verify their shape and surface features, microscopy analysis did not seem to be the most suitable technique; therefore, scanning electron microscope (SEM) analysis was exploited to analyze the different NP suspensions. As an example, in Figure 4 a photograph of NP-4 is reported. Spherical shape and smooth surface were observed at 20000× magnification and a bright contour probably due to ST-CS, absent in nanoparticles without coating, was also remarked. In a smaller magnification (data not shown), the sample appears homogeneously dispersed, without crystals nor aggregates; No significant difference in SEM analysis was registered in CURC-loaded NP (data not shown). 

**Figure 5:**
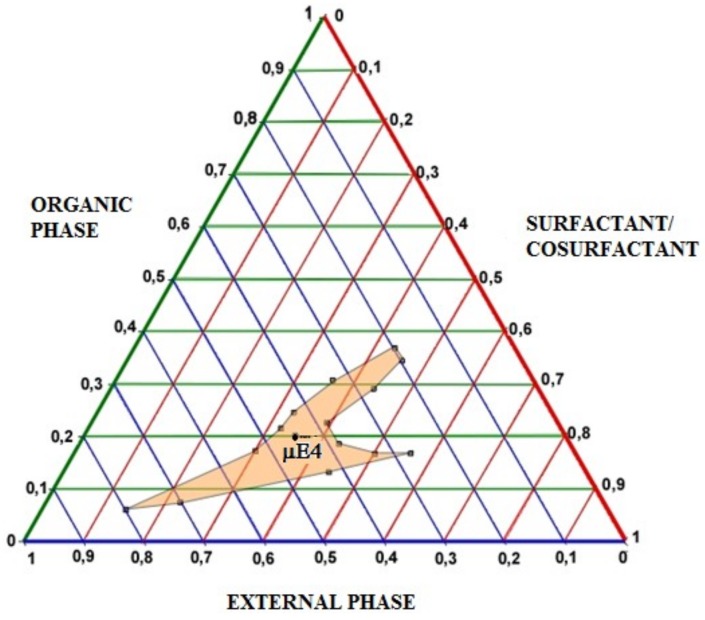
Pseudoternary phase diagram. EXTERNAL PHASE = s-water; ORGANIC PHASE = CHOL solubilized in s-BL, BA and ST-CS; SURFACTANT/COSURFACTANT = Epikuron^®^200-Cremophor^®^RH60-Taurocholic acid sodium salt at constant ratio.

CURC-free µE 4, that showed the lowest mean size and the best results concerning polydispersion, was characterized determining the area of existence by pseudoternary diagrams. In Figure 5 pseudoternary phase diagram is reported. The use of selected ingredients allowed to obtain a wide existence area in whose center µE-4 is placed.

**Figure 6:**
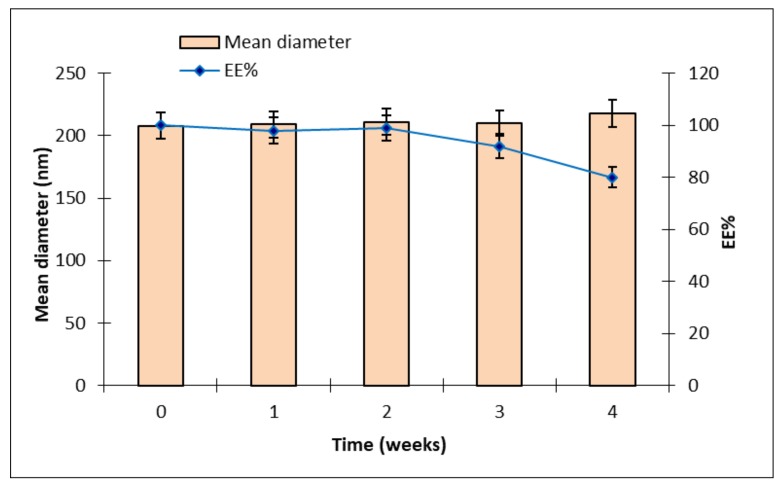
Over time, mean diameters and CURC EE% of CURC-NP-9 stored at 4 °C.

Mean sizes and EE% of CURC-NP-9, the best drug-loaded formulation, were monitored to evaluate NP stability over 4 weeks; results are reported in Figure 6. 

Mean diameter of CURC-NP-9 was stable throughout the period, while CURC EE% remained unchanged only for 2 weeks and then decreased up to 80% after 4 weeks; probably the NP structure was not enough rigid to keep CURC inside. Therefore, to preserve NP suspensions for long periods, further formulation studies and technological strategies such as freeze drying will be necessary.

**Figure 7:**
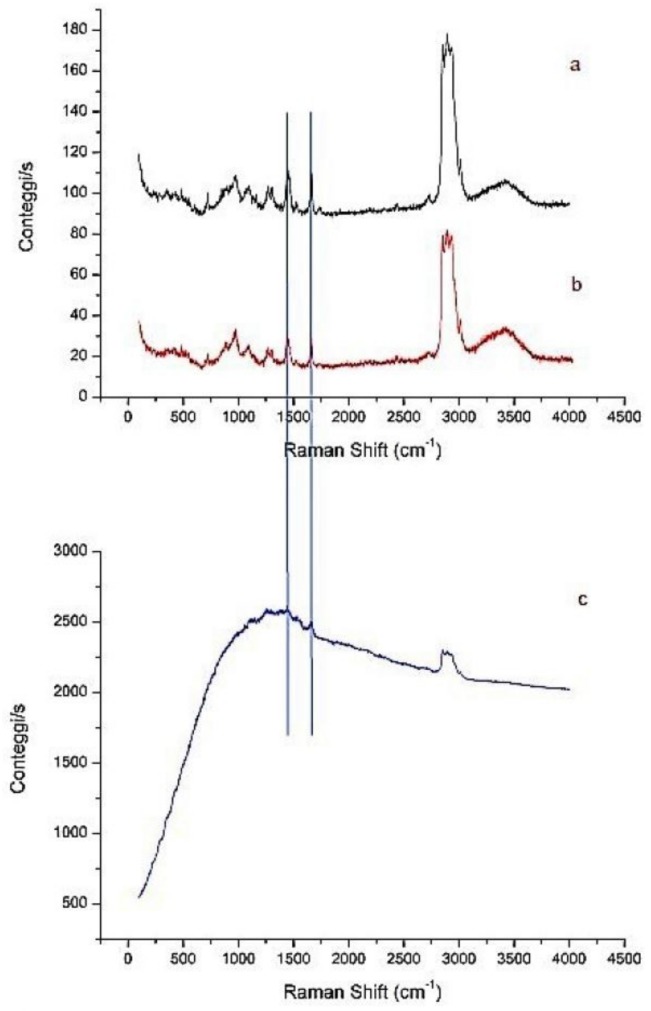
Raman spectra of NPs (**a**), NP-4 (**b**) and CURC-NP-9 (**c**), acquired using a 532 nm laser source.

To have general information about the NP composition, three different formulation were analyzed by Raman spectroscopy: NPs (without ST-CS coating), NP-4 and CURC-NP-9 (traces a, b and c in Figure 7).

Comparing the NPs spectrum with that of NP-4, it is possible to observe the same kind of vibrations attributable to aliphatic CH stretching and bending, as well as the C-C stretching. These oscillators are present both in NPs and in NP-4, so they are not diagnostics of the ST-CS presence. However, the small increment of the absorption peak related to OH oscillator is observed in the case of NP-4, which is consistent with an increasing of the number of this functional groups due to the presence of chitosan. The Raman spectrum of CURC-NP-9 is characterized by a large fluorescent peak due to the presence of curcumin.

**Figure 8:**
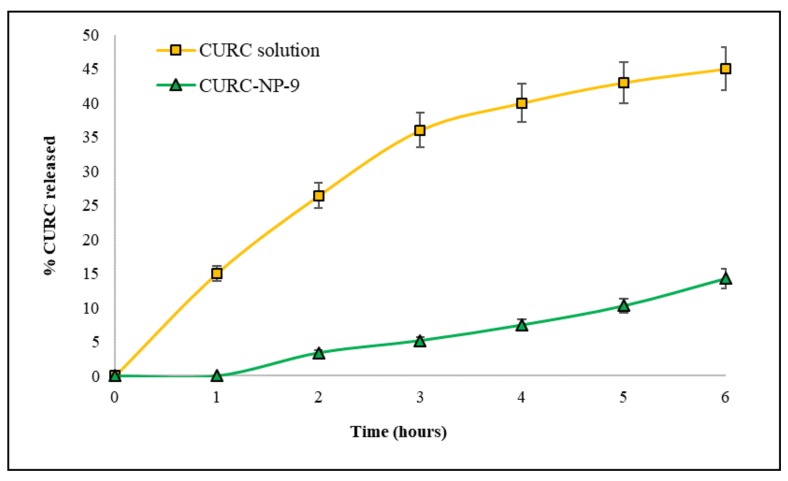
CURC release from solution of CURC in 10% PEG300 and from CURC-NP-9 suspension. Receiving medium was 10% PEG300 in water.

CURC release from CURC-NP-9 was studied and compared to that of the CURC solution. In Figure 8, profiles of both CURC releases, expressed as % CURC released versus time, are reported. 

The diffusion rate of CURC from the solution was fast (more than 40% released in 6 h); K_d_, calculated from the slope of the straight section of the curve was 0.078 cm h^−1^. CURC release from NP was slower and delayed compared to CURC solution: K_d_ was 0.015 cm h^−1^ and 1 h lag-time was observed, suggesting the presence of CURC inside the NP matrix. These results encourage the use of the developed NP as slow release CURC delivery systems.

**Figure 9:**
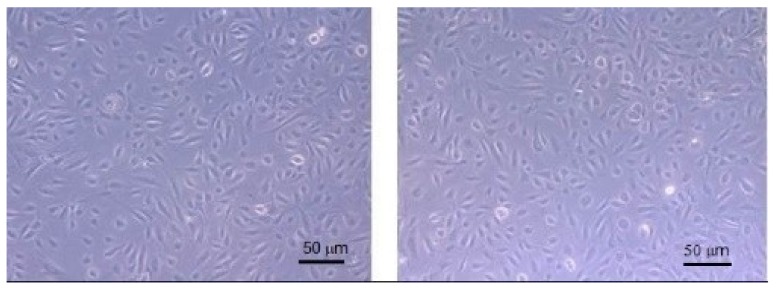
Human Umbilical Vein Endothelial Cells (HUVEC) before (left) and after (right) NP-4 treatment.

The following step of the research was the evaluation of NP safeness; therefore, NP-4 were tested on Human Umbilical Vein Endothelial Cells (HUVEC) to verify their cytotoxicity. Cells were cultured in the presence of titrated amount of NP-4 (1:100–1:1000) and the amount of viable cells was then assessed by the water-soluble tetrazolium salts assay (WST-1). Figure 9 shows HUVEC cells before and after NP-4 treatment: no cytotoxicity signals and no changes of morphology are present. 

**Figure 10:**
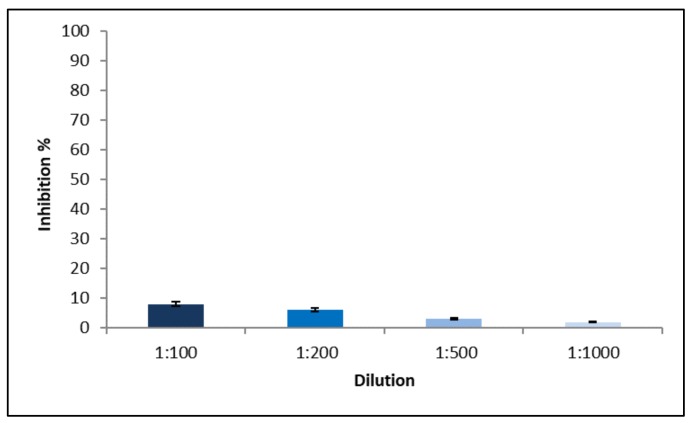
NP-4 cytotoxicity test on HUVEC at different dilutions.

This result was confirmed by WST1 test. Figure 10 shows that no cytotoxicity took place at all NP dilutions: all samples evidenced inhibition growth <10%. NP were composed mainly by physiological substances such as lecithin, CS and CHOL and, therefore, their lack of cytotoxicity was predictable; moreover, this result confirmed the usefulness of gel chromatography to remove surfactants and cosurfactants from the continuous phase of NP dispersion, which would probably be responsible for cytotoxicity. 

As underlined in the Introduction section, among numerous plant-derived nutraceuticals with anticancer properties, CURC is the most studied one. Its multiple anticancer effects were assessed by numerous studies in pancreatic cell lines and mice studies [17,18]. Moreover, CURC seems to be a very promising drug against pancreatic tumor so much that it was the object of Phase II Trials [19].

**Figure 11:**
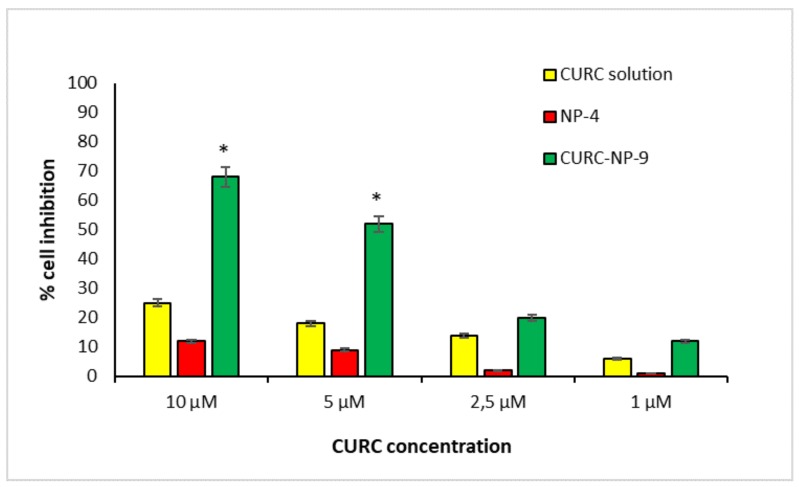
NP-4 in vitro cytotoxicity on PANC-1 cells determined by WST-1 assay. * *p* < 0.01.

Therefore, in the present study, PANC-1 cell line was used to compare the ability of CURC-NP-9 and free CURC solution to inhibit cancer cell growth. Figure 11 shows that CURC-NP-9 and free CURC inhibit in a concentration-dependent way the viability of the PANC-1 cell line. 

CURC solution, in accordance to literature data [20] shows a cell inhibition about 20% at the highest tested concentrations. CURC-NP-9 at the same concentrations seem to be more effective inhibiting cell vitality up to 70%. These results could be explained by assuming the internalization of CURC-NP-9 by PANC-1 cells and the following continuous release of CURC inside the cell; furthermore, the slow release of CURC from NP was previously noted in vitro (Figure 8). To confirm this hypothesis, further analysis, such as observation by confocal microscopy will be necessary. Moreover, no cytotoxicity was induced on PANC-1 cell line by NP-4, confirming the results obtained on HUVEC cell lines.

**Figure 12:**
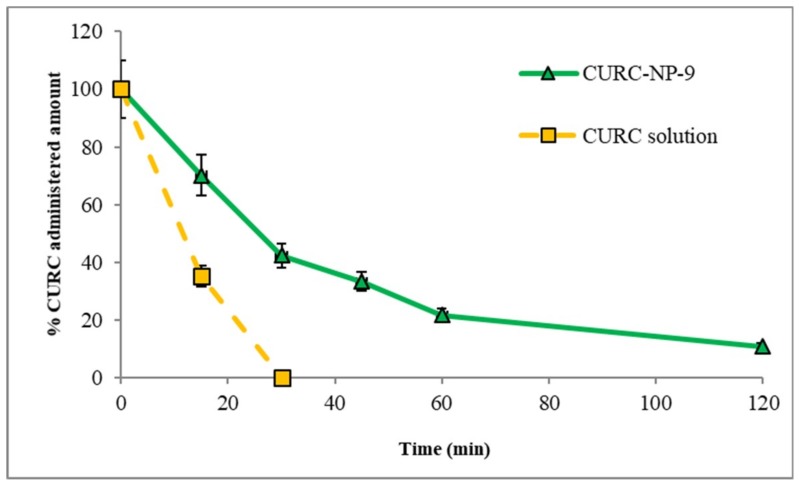
CURC blood concentration profiles vs time after intravenous administration of CURC-NP-9 and CURC solution in rats (2.0 mg/kg body weigh).

Studies of CURC pharmacokinetic and biodistribution were performed administering intravenously CURC solution and CURC-NP-9 at the dose of 2.0 mg/kg in rats. In Figure 12 pharmacokinetic profiles were reported. 

Blood profile studies demonstrated that the CURC solution was rapidly removed from bloodstream; in fact, already after 30 min, no trace of CURC in blood is revealed by the high-performance liquid chromatography (HPLC) analysis. A different profile was registered for CURC-NP-9. In this case, the decrease in blood CURC concentration was slower, and after 120 min almost 18% of the initial CURC concentration was still detected. This is probably due to a certain stealth property of NP, as the hydrophilic CS coating allows to minimize opsonization and, consequently, to prolong NP systemic circulation.

**Figure 13:**
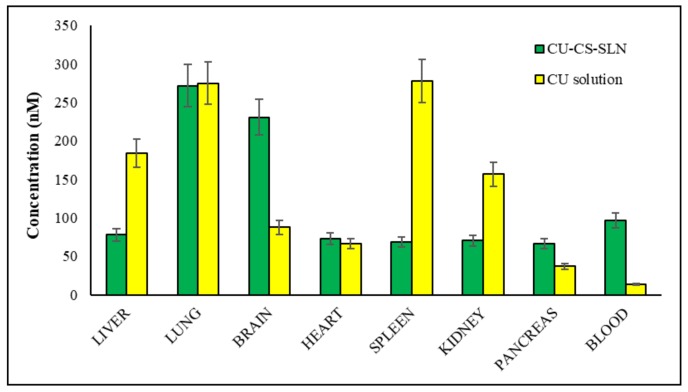
Biodistribution 30 min after intravenous administration of CURC-NP-9 and CURC solution in rats (2.0 mg/kg body weigh). *n* = 4.

**Figure 14:**
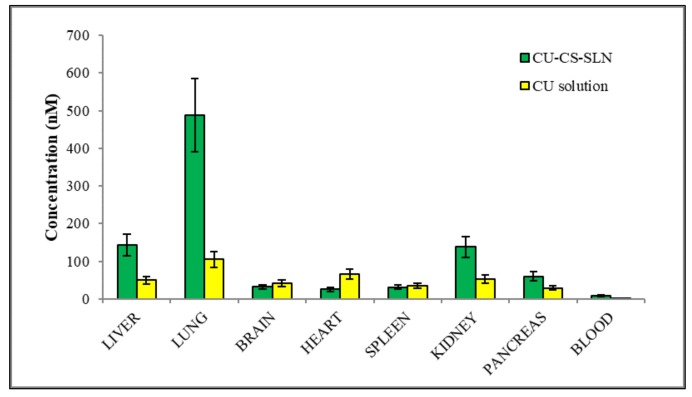
Biodistribution 60 min after intravenous administration of CURC-NP-9 and CURC solution in rats (2.0 mg/kg body weigh). *n* = 4.

Starting from these preliminary encouraging results, biodistribution studies were performed sacrificing rats 30 or 60 min after IV administration of CURC solution or CURC-NP-9 (Figure 13 and Figure 14).

Results reported in Figure 13 show that in the rat group receiving CURC solution, at 30 min after administration, significantly high concentrations of CURC were found in reticuloendothelial system (RES) organs such as spleen (278 nM), liver (184 nM) and kidney (157 nM). In rat group receiving CURC solution, CURC high concentration (275 nM) was also found in the lungs, according to literature data [21], due probably to the filtration of pulmonary capillary beds [22].

At the same after administration time, higher CURC blood, pancreas and brain concentrations were found in rat group receiving CURC-NP-9 than in that receiving CURC solution (according to pharmacokinetic data), while lower concentrations were registered in RES organs.

Quite surprising was that CURC at high concentration was recovered in the brain 30 min after IV administration: we assume that NP can cross blood-brain barrier (BBB) via an adsorptive mediated endocytosis. Indeed, it is well described in the literature [23] that electrostatic interaction of positively charged molecules with anionic endothelial cells cytoplasmic membrane overcomes the impeding action of the BBB and triggers the site specific transport of drug molecules to the brain. Moreover, in a previous work [24] the authors demonstrated that the BBB permeability, tested in vitro through hCMEC/D3 cells monolayer, showed a significantly increase in the permeation of Coumarin-6, used as model drug, when vehicled in solid lipid nanoparticles. 

The fast decrease in CURC concentrations in spleen, liver and kidney in rat group receiving CURC solution at 60 min after administration, suggests rapid elimination of CURC (Figure 14). 

On the contrary, in rat group receiving CURC-NP-9 a slight increase in CURC concentrations were noted in liver, kidney and lungs, indicating a certain accumulation probably also due to aggregation phenomena. It might also be supposed that NP could protect CURC from metabolism in this organ.

## 3. Materials and Methods

Epikuron^®^200 was purchased from ACEF (Fiorenzuola d’Arda, Italy), benzyl alcohol, propylene glycol, stearoyl chloride, cholesterol, butyl lactate from Fluka (Buchs, Switzerland), Fluorescein isothiocyanate, CS low viscosity, Sepharose^®^CL 4B, CURC, potassium chloride, Poly(ethylene glycol) 300, 2,3-bis[2-methoxy-4-nitro-5-sulphophenyl]-2H-tetrazolium-5-carboxanilide, crystal violet solution from Sigma (Dorset, UK), Cremophor^®^RH60 from BASF (Ludwigshafen am Rhein, Germany), Taurocholic acid sodium salt from ICN Biomedicals (Solon, OH, USA), potassium phosphate monobasic, potassium phosphate dibasic from Merck (Darmstadt, Germany), methanol, trimethylamine, sodium chloride, acetic acid and ethanol from Carlo Erba (Val De Reuil, France). Deionized water was obtained by a MilliQ system (Millipore, Bedford, MO, USA). 

### 3.1. Synthesis and Characterization of Stearoyl Chitosan (ST-CS)

ST-CS at different substitution degree was synthesized in order to obtain a lipophilic CS derivative. Freeze-dried commercial low viscosity chitosan (CS) was added to CH_3_CN into a dry flask; different amounts of stearoyl chloride (corresponding to 30% and 50% of the number of amino groups present on native CS) and triethylamine were added and the mixture was kept in an ultrasound bath at 25 °C for 4 h (Figure 1). Afterwards, the reaction solution was centrifuged for 15 min at 55,000 g; the supernatant was withdrawn and the precipitate washed thrice with water. Finally, the reaction product was freeze-dried to obtain a fine powder. 

ST-CS derivatives were characterized with NMR spectra performed using Jeol ECZ-R 600 MHz spectrometer equipped with a solid probe operating at 14 T at room temperature and spectral width of 10 KHz. 

An aliquot of ST-CS was suspended into CH_3_CN; FITC and potassium carbonate were then added to obtain a fluorescent ST-CS derivative (FITC-ST-CS). The mixture was kept 2 h in an ultrasound bath at 25 °C and then centrifuged for 15 min at 55,000 g. The precipitate was washed thrice with water to eliminate unreacted FITC and then freeze-dried.

### 3.2. ST-CS Coated Nanoparticles (ST-CS NP) Preparation

ST-CS NP were prepared using the innovative technique called “cold dilution of microemulsion” [25]. This technique involves the preparation of an O/W microemulsion (µE) using a partially water-soluble organic solvent as disperse oil phase. Following the dilution of µE with water, the solubilization of the organic solvent in water occurs, with the consequent NP precipitation. In our experimental conditions, butyl lactate (BL) was chosen as partially water-soluble organic solvent.

Different O/W µE were prepared using cholesterol (CHOL) dissolved in water-saturated BL (s-BL) as internal phase, BL-saturated water (s-water) as external phase, and various surfactants and cosurfactants. ST-CS was pre-solubilized in benzyl alcohol (BA) and then added in the dispersed phase. The resulting µE compositions are reported in Table 1. µE were diluted with water to obtain the precipitation of ST-CS NP. 

ST-CS NP suspensions were purified by gel chromatography using agarose cross-linked gel (Sepharose^®^ CL 4B) as stationary phase. Briefly, 1 mL ST-CS NP was introduced at the head of a 10 mL-column and eluted by gravity with hypertonic PBS (8.0 g/L NaCl, 0.2 g/L KCl, 1.44 g/L Na_2_HPO_4_ 2H_2_O, 0.24 g/L KH_2_PO_4_). Fractions of 1 mL each were collected. The opalescent fractions containing purified ST-CS NP were concentrated under nitrogen up to 1 mL final volume. 

To verify the effective presence of ST-CS on NP surface, FITC-ST-CS NP were prepared introducing FITC-ST-CS in µE1. 

### 3.3. Curcumin Loaded ST-CS NP (CURC-ST-CS NP) Preparation

µE reported in Table 1 were also prepared in the presence of CURC, that was solubilized with CHOL in s-BL; the continuous aqueous phase, surfactant and cosurfactant were then added to form a clear system. The final step was the precipitation of ST-CS NP obtained by µE water dilution and their purification by gel chromatography, as described in the previous paragraph. Gel chromatography was necessary to separate CURC-loaded particles from the free drug. 

### 3.4. Pseudo-Ternary Diagrams

CURC-free µE were characterized by pseudo-ternary phase diagrams construction at room temperature. Phase behavior of disperse systems was mapped on pseudo-ternary phase diagrams, obtained by titrating a series of surfactant/organic phase mixtures with s-water at room temperature. Constant ratios of Epikuron^®^200-Cremophor^®^RH60-Taurocholic acid sodium salt were used as surfactant, and CHOL solubilized in s-BL, BA and ST-CS as organic phase, s-water as aqueous phase. 

Appropriate amounts of surfactant and organic phase were weighed (200 mg) into glass ampules, shaken for sufficient time to reach equilibrium, and then progressively enriched by the aqueous phase, added dropwise. The amounts of added aqueous phase at which the transparent/opaque transition occurred were used to determine the phase domains. No attempt was made to distinguish between µE and discontinuous structures, and the domain of transparent, isotropic systems with mean diameter >5 nm was considered as the µE phase, while the domain of existence of turbid systems was classified as the emulsion phase. For the purposes of this study, a schematic representation was sufficient as a guide to follow the evolution of phase equilibrium. This experimental procedure was repeated for other surfactant to organic phase weight ratios and phase diagrams were constructed. 

### 3.5. ST-CS NP Characterization

ST-CS NP suspensions were observed by optical microscopy (Leica DM 2500, Solms, Germany) at 787 × magnification.

Shape and mean sizes of ST-CS NP were determined by scanning electron microscope (SEM) using a Stereoscan 410 (Leica, Wetzlar, Germany). Purified ST-CS NP suspensions were filtered through 220 nm membrane filter, washed with water to remove salts and then dried over vacuum. Filters were then deposited on copper stubs and samples were sputter coated with 15 nm graphite layer.

ST-CS NP sizes and polydispersity indexes (PDI) were determined by laser light scattering technique (LLS, Brookhaven, New York, USA). Size measurements were obtained at an angle of 90° at 25 °C using the intensity method. ST-CS NP suspensions were diluted with water or with 0.01 M KCl for size and Zeta-potential determination respectively.

CURC entrapment efficiency (EE%) was calculated as the ratio between CURC amount in ST-CS NP suspension after purification and that in the suspension before purification × 100. CURC EE% determination was performed diluting 50 µL both suspensions with 950 µL ethanol and injecting the obtained solutions in HPLC for CURC quantification.

### 3.6. CURC-ST-CS NP Stability

CURC-ST-CS NP sizes and CURC EE% of 4 °C-stored samples were monitored for 30 days to study NP physical stability and CURC entrapment stability. Mean sizes and CURC EE% determinations were carried out according to the previously described methods.

### 3.7. Curcumin In Vitro Release

In vitro release of CURC from CURC-ST-CS NP was determined using the non-equilibrium dialysis method [26] using a multicompartmental rotating cell system consisting of donor and receptor compartments of equal volume (1.5 mL) separated by a dialysis membrane (cut-off 14,000 Da). The receiving medium was 10% PEG300 in water. A solution of CURC in 10% PEG300 and purified CURC-ST-CS NP suspension (CURC-NP-9 of Table 2) were used as donor formulations. At fixed times, the receptor solution was tipped out and used for HPLC analysis and the cell was refilled with fresh receiving medium, obtained from sink conditions. Drug concentration was determined by HPLC. The results were evaluated as CURC apparent permeability constant (*Kd*_app_ [cm h^−1^]) calculated from the slope of the straight section of the curve (pseudo zero-order kinetics is assumed in this section), obtained by plotting the amount of diffused CURC vs time.

### 3.8. Raman Spectroscopy

Raman spectra were acquired with a Horiba Jobin Yvon HR800 Raman micro-spectrometer equipped with two alternative diffraction gratings, characterized respectively by 600 lines mm^−1^ and 1800 lines mm^−1^. The optical excitation was provided by a continuous 532 nm laser focused with a 100 × air objective. The excitation radiation was filtered out from the charge-coupled device (CCD) detection system by a narrow-band notch filter (Super Notch Plus 532 nm filter, 6.0 optical density, 10 nm spectral bandwidth). The employed magnification allowed to probe the entire volume of isolated NDs or small aggregates, since the laser spot is ~2 μm both in diameter and in focal depth. The spectrometer also allows controlling the laser power intensity in the 0.05–4.11 mW range, inserting different filters along the excitation optical path.

### 3.9. High-Performance Liquid Chromatography (HPLC) Analysis

HPLC analysis was performed using a LC9 pump (Shimadzu, Tokyo, Japan) with a Inertsil™ ODS-2 5 μm, 150 × 4.6 mm column and a C-R5A integrator (Shimadzu, Tokyo, Japan); mobile phase: CH_3_OH:H_2_O:CH_3_COOH 70:30:1 (flow rate 1 ml min^−1^); detector: UV λ = 450 nm (Shimadzu, Tokyo, Japan). Retention time was 5.0 min.

The limit of quantification, defined as the lowest CURC concentration in the curve that can be measured routinely with acceptable precision and accuracy, was 0.7 µmol/mL; the limit of determination, defined as the lowest detection limit, was 0.3 µmol/mL (signal to noise > 2.0).

### 3.10. HPLC Analysis of In Vivo Samples

The analysis of CURC in plasma and in homogenized organs was performed using a YL9100 system equipped with a YL9110 pump, a YL9101 vacuum degasser and a Shimadzu RF-10A fluorescence detector (Shimadzu, Tokyo, Japan), linked to YL-Clarity software for data analysis (Young Lin, Hogye-dong, Anyang, Korea). HPLC separation was performed on a LiChrospher^®^100 RP-18 5 μm 250 × 4.6 mm column and the mobile phase consisted of solvent A (3.5% *v/v* CH_3_COOH) and solvent B (CH_3_OH) delivered at 1 ml/min in isocratic conditions. For gradient elution, the starting mobile phase ratio was 60%A:40%B which was increased linearly in 10 min to 10%A:90%B and kept constant for 5 min. The analysis was monitored at λ_ex_ = 409 nm and λ_em_ = 509 nm.

### 3.11. In Vitro Cytotoxicity

To evaluate the extrinsic cytotoxicity of new ST-CS NP, in vitro tests were performed using HUVEC cell line (human umbilical vein endothelial cells) cultured in M199 medium with the addition of 20% heat-inactivated fetal calf serum (FCS), 100 U/mL penicillin, 100 μg/mL streptomycin, 5 UI/mL heparin, 12 μg/mL bovine brain extract and 200 mM glutamine at 37 °C in 5% CO_2_ humidified atmosphere. The purified ST-CS NP sample (NP-4 of Table 2) was opportunely diluted at 1:100, 1:200, 1:500, 1:1000 ratios. Such dilutions corresponded to those applied to CURC-loaded NP in further cytotoxicity studies of CURC on PANC-1 cell lines.

CURC-ST-CS NP cytotoxicity was determined using PANC-1 (pancreatic adenocarcinoma cell line) obtained from the American Type Culture Collection (ATCC, Manassas, VA) and grown as a monolayer culture in RPMI 1640 medium supplemented with 10% FCS, 2 mM l-glutamine and 100 units/ml penicillin/streptomycin at 37 °C in 5% CO_2_ humidified atmosphere.

The WST-1 assay is a colorimetric test based on the cleavage of a tetrazolium salt, MTS, by mitochondrial dehydrogenases to form formazan in viable cells. Cells (1x10^3^/well) were seeded in 96-well plates and incubated at 37 °C, 5% CO_2_ for 24 h. Then, they were treated with different concentrations of dimethyl sulfoxide (DMSO) CURC solution or CURC-ST-CS NP (CURC-NP-9 of Table 2, 1–10 µM) and with the same dilutions of empty ST-CS NP (-NP-4 of Table 2).

After 72 h-incubation, each well was added by 10 µL tetrazolium salt WST-1 solution (4-[3-(4-Iodophenyl)-2-(4-nitro-phenyl)-2H-5-tetrazolio]-1,3-benzene disulfonate) and after 2 h the amount of formazan was evaluated by spectrophotometric analysis at 450 nm. Controls (i.e., cells that had received no samples) were normalized to 100%, and the readings from treated cells were expressed as % of viability inhibition.

Eight replicates were used to determine each data point and five different experiments were performed.

### 3.12. Curcumin Blood Profile Study

CURC-CS-NP (CURC-NP-9 of Table 2) and CURC solution (2.0 mg/kg body weight) were administered through a catheter surgically positioned in the jugular vein of 4 male Wistar healthy rats (weight, 450 g). At scheduled times (5, 15, 30, 45, 60 and 120 min after administration), a rat blood aliquot was drawn.

200 μL CH_3_CN were added to 100 μL blood, vortexed and centrifuged for 15 min at 55,000× g. The supernatant was analyzed by in vitro HPLC method.

The procedures conformed to the Ethics Committee of University of Turin’s institutional guidelines on animal welfare (D. Lgs. 26/2014) as well as international Guidelines, and all efforts were made to minimize the number of animals and their discomfort (3R guidelines).

All experiments on animal models were performed according to an experimental protocol approved by the University Bioethical Committee, and authorized by the Italian Ministry of Health (authorization n. 0165, 17/03/2015).

### 3.13. Curcumin Biodistribution Study

CURC-ST-CS-NP (CURC-NP-9 of Table 2) and CURC solution (2.0 mg/kg body weight) were administered through a catheter surgically positioned in the jugular vein of male Wistar healthy rats (weight, 450 g). At scheduled times (30, 60 and 120 min after administration), rats were sacrificed by CO_2_-induced euthanasia; plasma was withdrawn and organs (liver, spleen, kidneys, pancreas, lungs, heart and brain) were removed surgically.

Each experiment was performed on 4 rats for both administered samples. Organs were homogenized with UltraTurrax^®^ (IKA, Staufen, Germany) for 5 min in water at a tissue concentration of 250 mg/mL; CURC was extracted with CH_3_CN from tissue homogenates and plasma. The supernatant obtained after centrifugation was analyzed by HPLC equipped with a fluorometric detector.

## 4. Conclusions

In this work a new nanoparticle production technique was successfully developed and CURC-ST-CS spherical nanoparticles were prepared. CURC, a natural molecule recently widely studied for its promising properties in different application fields, was chosen as a model drug because of its great bioavailability problems. Nanoparticles were coated with CS with the aim of protecting them from opsonization and prolonging their permanence in the blood; in particular, the use of a synthesized lipophilic derivative of CS permitted it to be firmly anchored to the lipid nanoparticle matrix.

The ST-CS-NP obtained have mean diameters in the range 200–250 nm, slightly positive Zeta potential, and high CURC entrapment efficiency. In vitro studies evidenced the lack of cytotoxicity of NP-4 and the increase of PANC-1 cells inhibition proliferation by CURC-NP-9.

Considering the results obtained monitoring blood profile overtime, the efficacy of CS coating in prolonging CURC-NP-9 permanence in blood compared to CURC solution was confirmed. Interesting results obtained from biodistribution studies evidenced an increase of CURC accumulation in brain and in pancreas when CURC was in nanoparticles compared to CURC solution. Furthermore, comparing these results with those previously obtained with nanoparticles prepared by the same technique but with a different excipient and without CS coating (data not shown), a lesser amount of CURC was founded in RES organs when the drug was entrapped in ST-CS-NP.

ST-CS-NP are, therefore, promising carriers for improving CURC biodistribution and to facilitate its application in therapy.

## Figures and Tables

**Table 1:** Microemulsions composition: empty (µE1–µE5) and 5 mg curcumin (CURC)-loaded (µE6–µE10).

Ingredients (mg)	µE 1 (6)	µE 2 (7)	µE 3 (8)	µE 4 (9)	µE 5 (10)
s-BL	74	74	74	74	74
CHOL	25	25	25	25	25
Benzyl alcohol	52			52	52
ST-CS	3.3	3.3	3.3	3.3	3.3
Epikuron^®^200	125	125	125	125	125
Taurocholic acid sodium salt (TC)	25	25	-	70	20
Cremophor^®^RH60	-	-	-	74	26
Tween^®^20	-	-	-	-	55
PEG 400	56	84	112	-	-
s-water	347	347	347	347	347

**Table 2:** Lipid nanoparticle (NP) and CURC-NP characteristics.

	Mean Diameter (nm)	Polydispersity	Zeta Potential (mV)	CURC EE%
NP-1	221.8 ± 1.1	0.170	+2.21 ± 0.41	-
NP-2	245.9 ± 1.7	0.200	+2.11 ± 0.32	-
NP-3	253.5 ± 1.4	0.210	+2.14 ± 0.35	-
NP-4	198.3 ± 1.1	0.090	+2.09 ± 0.46	-
NP-5	200.3 ± 1.6	0.101	+1.89 ± 0.24	-
CURC-NP-6	290.9 ± 2.1	0.180	+2.24 ± 0.42	50.7 ± 0.3
CURC-NP-7	330.6 ± 1.9	0.190	+2.31 ± 0.22	40.4 ± 0.6
CURC-NP-8	390.8 ± 2.1	0.250	+1.95 ± 0.36	33.2 ± 0.4
CURC-NP-9	190.6 ± 1.5	0.170	+2.10 ± 0.51	73.4 ± 0.3
CURC-NP-10	211.9 ± 1.5	0.160	+2.65 ± 0.54	60.6 ± 0.5

## Abbreviations

CS	Chitosan
ST-CS	Stearoyl chitosan
CURC	Curcumin
NP	Nanoparticles
BL	Butyl lactate
CHOL	Cholesterol
µE	Microemulsion
TC	Taurocholic acid sodium salt
BA	Benzyl alcohol
DMSO	Dimethyl sulfoxide
FITC	Fluorescein isothiocyanate
WST	Water-soluble tetrazolium salts
